# Comparison of small interfering RNA (siRNA) delivery into bovine monocyte-derived macrophages by transfection and electroporation

**DOI:** 10.1016/j.vetimm.2014.02.002

**Published:** 2014-04-15

**Authors:** Kirsty Jensen, Jennifer A. Anderson, Elizabeth J. Glass

**Affiliations:** Division of Infection & Immunity, The Roslin Institute and Royal (Dick) School of Veterinary Studies, University of Edinburgh, Easter Bush Campus, Midlothian EH25 9RG, UK

**Keywords:** Bovine, Macrophage, siRNA, Electroporation, Transfection

## Abstract

The manipulation of the RNA interference pathway using small interfering RNA (siRNA) has become the most frequently used gene silencing method. However, siRNA delivery into primary cells, especially primary macrophages, is often considered challenging. Here we report the investigation of the suitability of two methodologies: transient transfection and electroporation, to deliver siRNA targeted against the putative immunomodulatory gene Mediterranean fever (MEFV) into primary bovine monocyte-derived macrophages (bMDM). Eleven commercial transfection reagents were investigated with variable results with respect to siRNA uptake, target gene knock-down, cell toxicity and type I interferon (IFN) response induction. Three transfection reagents: Lipofectamine 2000, Lipofectamine RNAiMAX and DharmaFECT 3, were found to consistently give the best results. However, all the transfection reagents tested induced an IFN response in the absence of siRNA, which could be minimized by reducing the transfection reagent incubation period. In addition, optimized siRNA delivery into bMDM by electroporation achieved comparable levels of target gene knock-down as transient transfection, without a detectable IFN response, but with higher levels of cell toxicity. The optimized transient transfection and electroporation methodologies may provide a starting point for optimizing siRNA delivery into macrophages derived from other species or other cells considered difficult to investigate with siRNA.

## 1 Introduction

The discovery of the RNA inference (RNAi) pathway, where double-stranded RNA (dsRNA) post-transcriptionally silences gene expression (Fire et al., 1998) and the subsequent demonstration that this pathway could be utilized with short, exogenous RNA sequences (Elbashir et al., 2001) have revolutionized many areas of cellular research. The RNAi pathway is evolutionary conserved, being present in plants, fungi and animal cells, and has multiple functions; protecting cells from viral dsRNA and transposons, as well as regulating gene expression by the degradation of mRNA, translational repression and chromatin modifications (reviewed by Dykxhoorn and Lieberman, 2005). In the cell cytoplasm, endogenous microRNA (miRNA) or exogenous dsRNA are typically processed into 21–23 nucleotide short interfering RNA (siRNA) by the Dicer protein and integrated into the multi-subunit RNA-induced silencing complex (RISC). The RNA strands are separated and the antisense (guide) strand binds mRNA with complementary sequence, resulting in mRNA cleavage or translational repression, depending on the extent of sequence complementarity (reviewed by Shan, 2010).

The ability to harness the RNAi pathway, either by the uptake of synthetic siRNA into cells, or by the introduction of vectors that express short hairpin RNA (shRNA) that mimic miRNA precursors, allows the silencing of virtually any gene of any organism and has proved to be an elegant tool for the reverse genetic investigation of gene function. siRNA libraries are now available for humans and mice, which allow high throughput screening of thousands of genes simultaneously, which have expanded our knowledge and identified novel genes involved in cellular pathways and host–pathogen interactions (Zhou et al., 2008; Jayaswal et al., 2010). Since nearly any gene can be silenced the technology has great therapeutic potential, if the siRNAs can be delivered to the desired cells or organs, and several siRNA therapeutics are currently in phase II and III trials (reviewed by Whitehead et al., 2009). A major advantage of employing the RNAi pathway is that genes can be silenced in non-model species, where reagents are limited but sequence information is available. This is particularly true for farm animals, where genome sequencing efforts have resulted in large sequence resources, but there are limited reagents, e.g. antibodies, available and few transgenic animals. Gene silencing by siRNA are now routinely used in studies on cattle (Ma and Corl, 2012), sheep (Smith et al., 2012), chickens (Qi et al., 2013), pigs (Shi et al., 2013) and salmon (Kumari et al., 2013) using cell lines and some primary cells. However, there are difficulties introducing exogenous nucleotides, including siRNA, into certain primary cells, especially primary macrophages (reviewed by Zhang et al., 2009) and studies using siRNA to investigate macrophage genes lag behind those for other cell types. There are several reasons for this difficulty, which relate to the function of macrophages. Macrophages play several important roles in the immune system. They detect pathogens and danger signals and respond by phagocytosis and destruction of pathogens, antigen presentation and the secretion of immunological mediators. For this they express many enzymes that can degrade nucleotides and are primed to react rapidly and vigorously to the presence of foreign particles. Our research focuses on investigating the role of bovine macrophages during infection with various pathogens, e.g. *Theileria annulata* (Jensen et al., 2009). As part of an on-going project we wished to investigate the functional importance of macrophage expressed genes by targeted silencing of genes in primary bovine monocyte-derived macrophages (bMDM). To our knowledge the use of siRNA or shRNA to knock-down gene expression in primary bovine macrophages has not been reported before. Therefore we set about investigating methods to silence bovine macrophage genes utilizing the RNAi pathway. Viral vectors, e.g. lentiviruses, can integrate into the genomes of non-dividing cells, resulting in constitutive expression of shRNA and long-term gene silencing (reviewed by Zhang et al., 2009) and lentiviruses have successfully transduced shRNA into primary human macrophages (Lee et al., 2003). However, one of the functions of macrophages is to respond to the presence of viruses, which may affect a subsequent response to challenge with the pathogen being investigated. Therefore, our efforts have concentrated on optimizing transient gene silencing in bMDM using the less immunogenic siRNA.

There are two predominant techniques that have been successfully used to introduce siRNA into primary macrophages derived from other, non-bovine species; transient transfection (Zhang et al., 2005; Wati et al., 2007; Behmoaras et al., 2008; Lietzen et al., 2011) and electroporation (Wiese et al., 2010). Transient transfection involves the use of cationic lipids which interact with negatively charged RNA or DNA and form lipoplexes by electrostatic forces (reviewed by Zuhorn et al., 2007) which are taken up by endocytosis. Electroporation involves the application of an external electric field which temporarily increases cell plasma permeability, causing pores to form in the plasma membrane through which RNA or DNA can directly enter cells (Neumann et al., 1982). Here we report our investigation of siRNA gene silencing in bovine bMDM and describe the optimization of siRNA delivery by transient transfection and electroporation.

## 2 Materials and methods

### 2.1 Preparation of bovine monocyte-derived macrophages (bMDM)

Peripheral blood from three female Holstein-Friesian cattle, maintained at The Roslin Institute, was collected aseptically into blood bags containing the anticoagulant CPDA-1 (Sarstedt). All animals were clinically normal and all experimental protocols were authorized under the UK Animals (scientific procedures) Act, 1986. In addition, The Roslin Institute's Animal Welfare and Ethics Committee (AWEC) ensure compliance with all relevant legislation and promote the adoption and developments of the 3Rs (reduction, replacement, refinement). Peripheral blood mononuclear cells (PBMC) were separated by density gradient centrifugation as described previously (Jensen et al., 2006). The PBMC were resuspended in RPMI-1640 medium without serum (Invitrogen) at 5 × 10^6^ cells/ml and initially cultured for 2 h at 37 °C, after which time the medium and non-adhered cells were replaced with RPMI-1640 supplemented with 20% foetal bovine serum (FBS), 4 mM l-glutamine and 50 μM β-mercaptoethanol. The adhered cells were cultured for 7 days at 37 °C, with a change of medium on day 4. During this time the monocytes within the PBMC were observed to develop macrophage morphology and the up-regulation of the macrophage markers; CD64, CD68, v-maf musculoaponeurotic fibrosarcoma oncogene homolog (avian) (MAF), macrophage scavenger receptor 1 (MSR1), scavenger receptor class B, member 2 (SCARB2) and triggering receptor expressed on myeloid cells 2 (TREM2), was detected by RT-PCR (see Supplementary File).

After 7 days the adhered cells were vigorously washed three times with PBS to remove the remaining non-adhered cells. The adherent cells were removed from the plates by incubation with TrypLE Express (Invitrogen) and then resuspended in RPMI-1640 supplemented with 20% FBS. The purity of the macrophage population was assessed by flow cytometry, using a mouse anti-bovine SIRPA (CD172α) antibody directly conjugated with RPE-Cy5 (AbD Serotec: Cat. No. MCA2041C), which confirmed that the macrophage purity exceeded 90% (see Supplementary File). All flow cytometry analysis was carried out on a CyAn flow cytometer (Beckman Coulter) using the Summit software.

### 2.2 siRNA duplexes

The AllStars negative control siRNA, which has no homology to any known mammalian gene, labelled with fluorescein isothiocyanate (FITC) (Qiagen) was used to assess the efficiency of siRNA uptake and as the negative control siRNA in the knock-down experiments. As part of on-going studies, siRNA were designed for the inflammasome-associated bovine Mediterranean fever (MEFV), using the RefSeq sequence XM_002706315. The siRNA were designed by Sigma–Aldrich and three siRNA were purchased and tested. All three siRNA induced target gene knock-down and the results for MEFV#3 (5′-GTTGCTTAATAAATCCTTA-3′) are described here. Stocks (20 μM) were prepared of each siRNA, which were aliquotted and stored at −20 °C.

### 2.3 Transfection

The efficacy of eleven transfection reagents to promote uptake of the FITC-labelled siRNA was investigated. The bMDM were seeded at 1 × 10^5^ cells/well in 24 well plates. After 3 h incubation at 37 °C, during which time the bMDM had adhered to the plastic, the cells were transfected with 50 nM siRNA. The transfection reagents; HiPerFect (Qiagen), INTERFERin (Polyplus), Lipofectamine RNAiMAX (Invitrogen), Lipofectamine 2000 (Invitrogen), DharmaFECT 1, DharmaFECT 2, DharmaFECT 3 and DharmaFECT 4 (Dharmacon), siPORT Amine (Ambion), X-tremeGENE (Roche) and N-TER (Sigma–Aldrich) were used according to the manufacturers’ instructions. After 16 h incubation at 37 °C the bMDM were harvested and analyzed by flow cytometry.

To investigate the efficacy of each siRNA to knock-down expression of the target genes, the bMDM were seeded at 2 × 10^5^ cells/well in 24 well plates and, after 24 h incubation at 37 °C, were transfected with transfection reagent alone or with 50 nM siRNA. The transfection reagents were used at the optimum conditions determined by the previous experiments. To investigate any response of bMDM to the transfection reagents the bMDM were either incubated at 37 °C for 48 h or the medium was changed after 24 h and the bMDM cultured for a further 24 h before being harvested and the RNA extracted. To investigate MEFV knockdown, bMDM were incubated at 37 °C for 24 h, then the medium was replaced and bMDM were cultured for an additional 24 h. To stimulate MEFV expression bMDM were activated with 100 ng/ml *Escherichia coli*-derived lipopolysaccharide (LPS) (Sigma) and bMDM were harvested and RNA extracted 2 h post activation.

### 2.4 Electroporation

The ability of electroporation to deliver FITC-labelled AllStar negative control siRNA (Qiagen) into bMDM was initially investigated and the protocol optimized. Two buffers were tested; Opti-MEM I (Invitrogen) and siPORT siRNA electroporation buffer (Ambion). The siRNA was transferred into 1 mm electrode gap cuvettes and either buffer was added to give a total volume of 50 μl. The bMDM suspension in either buffer, at 4 × 10^7^ cells/ml, was added to the cuvette and mixed by pipetting to give a final volume of 100 μl, containing 2 × 10^5^ cells and 1 μM siRNA. The cuvette was immediately pulsed at a range of voltage and capacitance levels using a Gene Pulser electroporator (BioRad). The electroporated bMDM were incubated at 37 °C for 10 minutes before being transferred into 24 well plates and the volume increased to 0.5 ml/well. After 16 h incubation at 37 °C the bMDM were harvested and analyzed by flow cytometry.

The electroporation of target gene siRNA was carried out using a scaled up version of the optimized conditions. bMDM were resuspended at 1.2 × 10^7^ cells/ml in Opti-MEM I and electroporated with 3 μM siRNA at 300 V and 25 μF. After 10 min incubation at 37 °C the bMDM were transferred into 6 well plates and the volume increased to 4 ml/well. Unless stated otherwise, the bMDM were incubated at 37 °C for 48 h before being harvested and the RNA was extracted.

### 2.5 Quantification of siRNA uptake and cytotoxicity

After 16 h incubation at 37 °C the bMDM transfected or electroporated with FITC-labelled siRNA were harvested using TrypLE Express, washed with RPMI-1640 supplemented with 20% FBS and resuspended in 1 ml PBS supplemented with 0.5% FBS. Cell toxicity was measured by staining the cells with 1 μM SYTOX Blue dead cell stain (Invitrogen) according to the manufacturer's protocol. The bMDM were analyzed using a CyAn flow cytometer (Beckman Coulter), the bMDM population was gated, by size and complexity (see Supplementary File), and the percentage of FITC labelled and SYTOX Blue labelled bMDM was measured.

### 2.6 Quantitative (q)RT-PCR analysis of target gene knock-down and interferon response

Total RNA was extracted from the bMDM samples using the RNeasy Mini Kit (Qiagen) according to the manufacturer's instructions with on-column DNase digestion. The quality and quantity of the resulting RNA was determined by gel electrophoresis and NanoDrop ND-1000 spectrophotometer (Thermo Scientific). First strand cDNA was reverse transcribed from 0.1 to 0.5 μg total RNA using oligo(dT) primer and Superscript II (Invitrogen) according to the manufacturer's instructions. The resulting cDNA was diluted 1:25 for all genes. The mRNA levels of MEFV and selected type I interferon (IFN)-response genes were quantified by qPCR using the FastStart Universal SYBR Green mastermix (Roche) as described previously (Jensen et al., 2009). Oligonucleotides were designed for the target gene MEFV and the IFN-response gene interferon-induced protein with tetratricopeptide repeats 1 (IFIT1) using Primer3 (Rozen and Skaletsky, 2000) and Netprimer (Biosoft International) software. The sequences of the MEFV oligonucleotides are 5′-GGACCCCTCAATCCAGAAAT-3′ and 5′-GATGCTCCCCAATCATCATC-3′. The sequences of the IFIT1 oligonucleotides are 5′-GCTGCCAAGTTTTACCGAAG-3′ and 5′-CAAAGCCCTGTCTGGTGATG-3′. The relative quantities of mRNA were calculated using the method described by Pfaffl (2001), using the qRT-PCR results for chromosome alignment maintaining phosphoprotein 1 (CHAMP1), previously known as Chromosome 13 open reading frame 8, to calculate differences in the template RNA levels and thereby standardize the results for the genes of interest (Jensen et al., 2009).

## 3 Results and discussion

### 3.1 Delivery of siRNA by transfection reagents

Liposome based transfection reagents provide a simple and cost-effective method to introduce siRNA into cells and is therefore the method of choice for many researchers. Successful silencing of target genes in non-bovine primary macrophages with siRNA delivered by transient transfection has been reported previously (Zhang et al., 2005; Wati et al., 2007; Behmoaras et al., 2008; Lietzen et al., 2011).

#### 3.1.1 Screening of transfection reagents

**Fig. 1:**
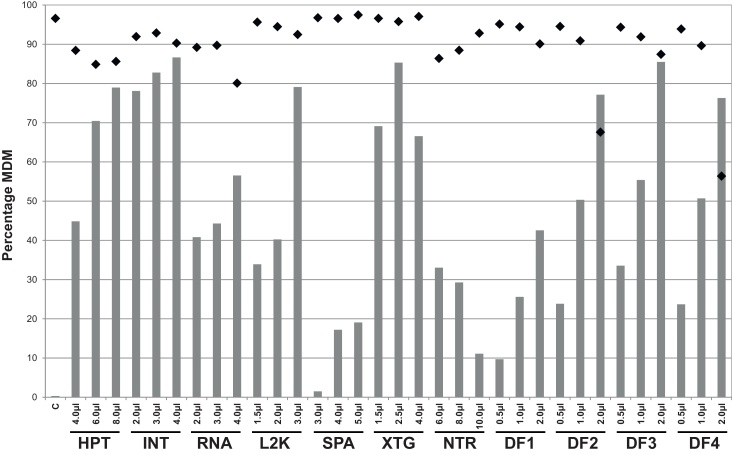
Comparison of the efficacy of eleven commercially available reagents to transfect siRNA into bovine monocyte-derived macrophages (bMDM). bMDM were transfected with 50 nM FITC-labelled siRNA using three different concentrations of the transfection reagents HiPerFect (HPT), INTERFERin (INT), Lipofectamine RNAiMAX (RNA), Lipofectamine 2000 (L2K), siPORT Amine (SPA), X-tremeGENE (XTG), N-TER (NTR) and DharmaFECT 1 (DF1), DharmaFECT 2 (DF2), DharmaFECT 3 (DF3) and DharmaFECT 4 (DF4) following the manufacturer's instructions. In addition bMDM were cultured with 50 nM FITC-labelled siRNA in the absence of transfection reagents as a control (C). After 16 h the percentage of bMDM transfected with FITC-siRNA (grey bars) and the percentage of viable bMDM (black diamonds) were measured by flow cytometry. The result is representative of three repeat experiments using bMDM from different animals.

We initially tested the suitability of eleven commercially available transfection reagents to promote siRNA uptake by bMDM. The preliminary experiments investigated the efficacy of three concentrations of each transfection reagent, within the range suggested by the manufacturers, to promote the uptake of a FITC-labelled non-target control (NTC) siRNA and the levels of cell toxicity that were induced. The efficacy of the eleven transfection reagents varied considerably, with the maximum percentage of bMDM taking up siRNA ranging between 19% and 86% cells with the different transfection reagents (Fig. 1). The reagents siPORT Amine, N-TER and DharmaFECT 1 failed to promote uptake of significant amounts of siRNA at the three different concentrations. The use of DharmaFECT 2 and DharmaFECT 4 resulted in siRNA up-take by a high proportion of bMDM, but caused considerable cell toxicity. The remaining transfection reagents: HiPerFect, INTERFERin, Lipofectamine 2000, Lipofectamine RNAiMAX, X-tremeGENE and DharmaFECT 3 promoted good up-take of siRNA by bMDM, without causing significant cell death. The levels of siRNA up-take were observed to increase with increasing levels of the transfection reagents, except for X-tremeGENE and N-TER. However, this enhanced up-take was usually associated with increased cell toxicity.

#### 3.1.2 Investigation of potential off-target effects of transfection reagents

**Fig. 2:**
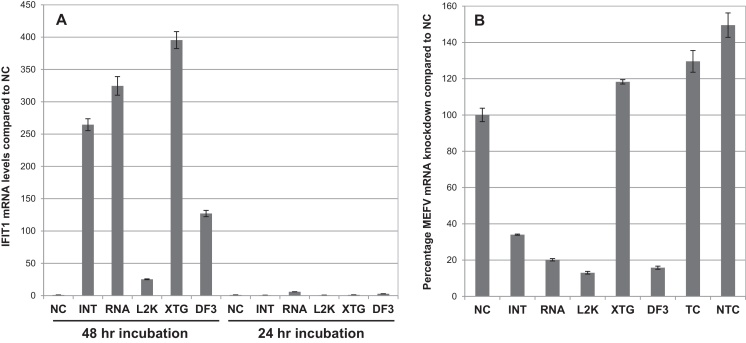
Transient transfection reagents induce a type I interferon (IFN)-response in bovine monocyte-derived macrophages (bMDM) in the absence of siRNA. The siRNA uptake efficacy and off-target effects of five transfection reagents, INTERFERin (INT), Lipofectamine RNAiMAX (RNA), Lipofectamine 2000 (L2K), X-tremeGENE (XTG) and DharmaFECT 3 (DF3) were investigated. (A) bMDM were cultured with pre-determined concentrations of the transfection reagents in the absence of siRNA for 48 h (48 h incubation) or the medium was changed 24 h post transfection reagent treatment and cultured for a further 24 h (24 h incubation). The mRNA levels of the type I IFN-response gene interferon-induced protein with tetratricopeptide repeats 1 (IFIT1) were measured by qRT-PCR. The results are expressed as the IFIT1 mRNA fold difference compared to that measured in untreated cells (NC). Error bars denote the variation between three technical replicates. (B) bMDM were transfected with MEFV siRNA using the five transfection reagents for 24 h before the medium was replaced. MEFV mRNA levels in bMDM transfected with (NTC) and without (TC) non-target control siRNA using Lipofectamine RNAiMAX were also investigated. After 48 h the bMDM were activated with 100 ng/ml LPS for 2 h. The results are expressed as the percentage MEFV mRNA detected compared to that measured in NC. Error bars denote the variation between three technical replicates. The results are representative of three repeat experiments using bMDM from different animals.

The results described above illustrate that siRNA are readily taken up by bMDM using certain transfection reagents. However, the suitability of transfection reagents also depends on the absence of the induction of off-target effects. The potential off-target effects induced by five transfection reagents: INTERFERin, Lipofectamine 2000, Lipofectamine RNAiMAX, X-tremeGENE and DharmaFECT 3, were investigated. These transfection reagents were selected for further study on the basis of their combined performance in terms of siRNA uptake and the cell toxicity induced (Fig. 1). bMDM were cultured with each transfection reagent, at the optimal concentration for siRNA uptake, for 48 h without siRNA. This time-point was chosen because several investigated siRNA induced maximal gene-silencing at this time in previous optimization experiments (data not shown). The expression of known type I IFN-response genes was then measured at the mRNA level by qRT-PCR. All five transfection reagents induced the up-regulation of IFIT1, ranging from 25-fold for Lipofectamine 2000 to 395-fold up-regulation with X-tremeGENE (Fig. 2A – 48 h incubation). Similar results were also measured for other type I IFN-response genes; interferon induced transmembrane protein 1 (IFITM1), myxovirus (influenzavirus) resistance 1, interferon-inducible protein (MX1) and 2′,5′-oligoadenylate synthetase 1 (OAS1) (data not shown). Off target transcriptional effects caused by the transfection reagents alone have been reported previously (Yoo et al., 2006; Tagami et al., 2008; Lacaze et al., 2009). In the absence of siRNA, Lipofectamine 2000 induced differential expression of over 500 genes in murine bone marrow derived macrophages, including many classic type I IFN response genes (Lacaze et al., 2009). Interestingly, if the medium was changed 24 h post treatment with the transfection reagents and the bMDM were cultured for a further 24 h, the IFN-response was not observed in bMDM (Fig. 2A – 24 h incubation). The maximum fold increase in IFIT1 mRNA that was measured was 6-fold induced by Lipofectamine RNAiMAX. Therefore the transfection reagents induce a response in bMDM, but this appears to be quickly dampened down by removing the stimulus. However, using a similar protocol 92 genes were still differentially expressed in human fibrosarcoma cells (HT1080) 24 h after Lipofectamine 2000 was removed from the cultures (Tagami et al., 2008).

#### 3.1.3 Target gene knock-down using transfection reagents

siRNA uptake does not guarantee target gene knock-down (Lundberg et al., 2007; Metwally et al., 2012) and therefore the ability of the five transfection reagents to promote target gene knock-down using siRNA was investigated. bMDM were treated with optimal concentrations of transfection reagent and siRNA against the inflammasome-associated molecule MEFV, the function of which we are currently investigating. The bMDM were cultured with transfection reagents and siRNA for 24 h and then the medium was replaced and the bMDM were cultured for a further 24 h. Levels of MEFV mRNA are below detectable levels in resting bMDM and therefore bMDM were stimulated with LPS to induce MEFV expression and levels of MEFV mRNA were measured by qRT-PCR 2 h post activation. The delivery of MEFV specific siRNA by the transfection reagents INTERFERin, Lipofectamine 2000, Lipofectamine RNAiMAX and DharmaFECT 3 reduced the MEFV mRNA levels to between 13.0 and 34.0% of that measured in activated non-siRNA treated cells (NC) (Fig. 2B). X-tremeGENE consistently failed to knock-down mRNA for MEFV (Fig. 2B) or other target genes (data not shown), illustrating that up-take of siRNA does not equate with target gene knock-down. MEFV gene silencing did not affect mRNA levels of the house-keeping gene glyceraldehyde-3-phosphate dehydrogenase (GAPDH) or those of other investigated genes (data not shown). The transfection reagents alone and the NTC siRNA did not diminish MEFV mRNA levels, but actually enhanced them. The MEFV mRNA levels measured in Lipofectamine RNAiMAX treated bMDM with (NTC) or without (TC) non-target control siRNA are illustrated in Fig. 2B. On average MEFV levels were 130% higher in TC than NC, although this was not statistically significant (*P* = 0.08). However, MEFV mRNA levels in NTC samples were statistically significantly higher than in NC samples, on average 150% greater (*P* < 0.05). Similar results were obtained for the other transfection reagents. This is evidence that 24 h after their removal transfection reagents are affecting bMDM biology, by modifying the immune response induced by a second stimuli. Furthermore, the scrambled siRNA, which has no homology to any known mammalian gene, is having an additional effect on MEFV mRNA expression.

Specific anti-bovine MEFV antibodies are not commercially available and constructing one was beyond the scope of this work. Unfortunately attempts to detect bovine MEFV using three different anti-human MEFV polyclonal antibodies, which due to MEFV sequence conservation are predicted to cross-react with bovine MEFV, failed. Therefore we have been unable to directly confirm knock-down of MEFV at the protein level. However, we have shown that knocking-down bovine MEFV, which regulates inflammasome activity (Seshadri et al., 2007), affects IL1B protein release (Jensen et al., in prep). This result is in agreement with studies knocking down human MEFV using a similar experimental design (Seshadri et al., 2007), providing indirect evidence that the MEFV siRNA is reducing bovine MEFV at the protein level.

Amongst the eleven transfection reagents investigated, Lipofectamine 2000, Lipofectamine RNAiMAX and DharmaFECT 3 consistently gave the best results, in terms of siRNA uptake and target gene knockdown with minimal cell toxicity and type I IFN response induction. Therefore, all three transfection reagents appear suitable for use in bMDM. Lipofectamine 2000 and RNAiMAX have successfully been used for siRNA gene silencing in murine bone-marrow derived macrophages (Lacaze et al., 2009) and human monocyte-derived macrophages (hMDM) (Wati et al., 2007) respectively. However, in another study siRNA were delivered into hMDM using DharmaFECT 1 (Behmoaras et al., 2008) which was unsuitable for use in bMDM, resulting in low levels of siRNA uptake. Furthermore, siPORT Amine was found to be the best transfection reagent for siRNA delivery into porcine alveolar macrophages (Zhang et al., 2005), which failed to facilitate siRNA uptake by bMDM. Therefore, there is considerable variation in the efficacy of transfection reagents to deliver siRNA into macrophages derived in different ways and from different species.

### 3.2 Delivery of siRNA by electroporation

The majority of cationic liposome-delivered siRNAs enter cells by endocytosis (Lu et al., 2009). The resulting off-target immune response of cells to siRNA and transfection reagents has been associated with their presence in the endosome (Sioud, 2005; Yoo et al., 2006). An alternative approach to introduce siRNA into cells is the use of electroporation, the application of an external electrical field which temporarily increases cell plasma permeability (Neumann et al., 1982), thus delivering siRNA directly into the cell cytoplasm. Using this technique can prevent the sequence-specific immune response induced by siRNA (Sioud, 2005) and has successfully been used to introduce siRNA into primary murine bone-marrow derived macrophages, resulting in target gene knock-down (Wiese et al., 2010). Therefore, the suitability of this approach to deliver siRNA for targeted gene knock-down in bMDM was investigated.

#### 3.2.1 Optimization of siRNA uptake by electroporation

**Fig. 3:**
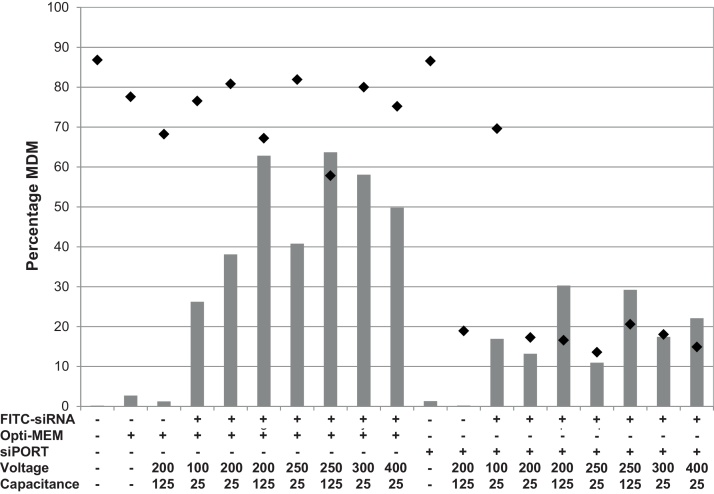
Optimization of siRNA uptake by electroporation. The ability of bovine monocyte-derived macrophages (bMDM) in different buffers, Opti-MEM and siPORT siRNA electroporation buffer, to take up 1 μM FITC-labelled siRNA after electroporation at various voltage and capacitance settings was compared. After 16 h the percentage of bMDM transfected with FITC-siRNA (grey bars) and the percentage of viable bMDM (black diamonds) were measured by flow cytometry. The result is representative of three repeat experiments using bMDM from different animals.

Initially cell viability and uptake of FITC-labelled NTC siRNA were optimized. The cells were tested in two media, Opti-MEM I and siPORT siRNA electroporation buffer, and the voltage and capacitance settings were adjusted. The different conditions altered the uptake of siRNA and cell viability considerably (Fig. 3). The siPORT siRNA electroporation buffer was not suitable for use with bMDM, its use resulted in over 80% cell death in all electroporation conditions tested. In contrast, the use of Opti-MEM I reduced the level of cell toxicity, although this varied considerably depending on the electroporation conditions. The highest level of siRNA uptake, exceeding 60%, was observed at voltage 200 V and 250 V with capacitance set at 125 μF, although these maximal values were lower than observed with the transfection reagents (Fig. 1). However, these conditions induced relatively high levels of cell toxicity, killing over 30% bMDM. Taking into account the siRNA uptake and cell toxicity induced, the electroporation conditions 300 V and 25 μF were optimal (Fig. 3) and these settings were used in the later experiments.

#### 3.2.2 Investigation of target gene knock-down and potential off-target effects with electroporation

**Fig. 4:**
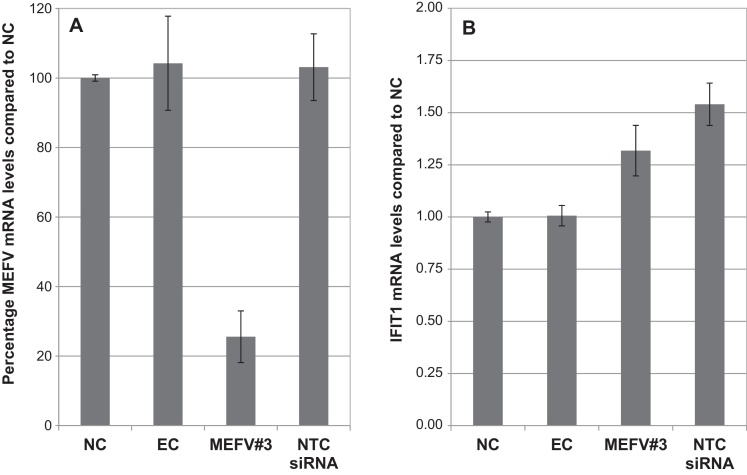
Electroporation is an effective method for siRNA uptake in bovine monocyte-derived macrophages (bMDM) without inducing a type I interferon (IFN) response. bMDM were electroporated without siRNA (EC), with MEFV siRNA or non-target control siRNA (NTC siRNA). (A) After 48 h the electroporated bMDM were activated with 100 ng/ml LPS for 2 h. The results are expressed as the percentage MEFV mRNA detected compared to that measured in untreated cells (NC). Error bars denote the variation between three technical replicates. (B) After 48 h cells were harvested and mRNA levels of the IFN-response gene interferon-induced protein with tetratricopeptide repeats 1 (IFIT1) were measured by qRT-PCR. The results are expressed as the IFIT1 mRNA fold difference compared to that measured in NC. Error bars denote the variation between three technical replicates. The results are representative of three repeat experiments using bMDM from different animals.

The ability of electroporated siRNA to induce target gene knock-down in bMDM was investigated. bMDM were electroporated with MEFV siRNA, NTC siRNA or in the absence of siRNA and MEFV mRNA levels were measured 2 h post activation of bMDM incubated with siRNA for 48 h. MEFV mRNA levels were reduced by 75% in bMDM electroporated with MEFV siRNA (Fig. 4A). This is comparable to levels measured in bMDM transfected with this siRNA (Fig. 2B). Neither the electroporation process nor the presence of the siRNA was associated with any change in IFIT1 levels (Fig. 4B) or any other IFN-response gene investigated (data not shown). Therefore electroporation is a suitable alternative to the use of transfection reagents to deliver siRNA into bMDM.

### 3.3 Conclusions

The results reported here demonstrate that, contrary to the dogma that primary macrophages are difficult to transfect, primary bMDM can be electroporated or transfected with siRNA resulting in good levels of target gene silencing. The methodologies described above have now been used to successfully silence nine genes in bMDM (manuscripts in preparation). It is hoped that these methodologies will provide a starting point for optimizing siRNA use in primary macrophages from other species and other primary cells which are regarded as being hard to transfect, e.g. dendritic cells. Several of the tested transfection reagents appear suitable for use: DharmaFECT 3, Lipofectamine 2000 and RNAiMAX. Electroporated siRNA silenced the MEFV gene to a comparable level as transfected siRNA, but the procedure resulted in more cell death and the remaining cells were less robust than transfected cells in down-stream activation studies (data not shown). The transfection protocols allow siRNA uptake by adhered cells and therefore it is much easier to reapply siRNA to bMDM, thereby extending the period of gene silencing, than by electroporation. The choice of transfecting or electroporating siRNA into cells depends on the individual experiments. The fragility of electroporated cells to challenge means that the use of transfection reagents is more suitable than electroporation for our work investigating the role of host macrophage genes in the response to infection. However, the increased expression of MEFV with transfection reagent treatment illustrates that both methodologies do affect macrophages and highlights the importance of the inclusion of suitable controls in siRNA experiments.

## Conflict of interest statement

The authors declare that no conflicting financial interests exist.
