# Supplementary material for: Comparison of small interfering RNA (siRNA) delivery into bovine monocyte-derived macrophages by transfection and electroporation
Source: Vet Immunol Immunopathol. 2014 Apr 15;158(3-4):224–32. doi: 10.1016/j.vetimm.2014.02.002 (PMC3988888; doi:10.1016/j.vetimm.2014.02.002)
Supplement: Supplementary file 1 [file mmc1.docx]

**Supplementary File**

**Section 1: Validation of differentiation state of bovine monocyte-derived macrophages (bMDM)**

Transcriptional markers of human monocyte to macrophage differentiation were identified from the literature (Martinez et al., 2006; Lehtonen et al., 2007; Liu et al., 2008), including cell surface molecules, e.g. Fc fragment of IgG, high affinity Ia, receptor (FCGR1A), and transcription factors, e.g. CCAAT/enhancer binding protein (C/EBP), delta (CEBPD). Oligonucleotide primers were designed for bovine homologues of eight identified markers using Primer3 (Rozen & Skaletsky, 2000) and Netprimer (Biosoft International) software (Table S1). For comparison with bMDM, monocytes were isolated from bovine peripheral blood under cold conditions as described previously (Jensen et al., 2006), except that monocytes were isolated using microbeads directly conjugated with anti-human CD14 antibody (Miltenyi Biotec). Monocytes were immediately harvested after purification for RNA extraction. Total RNA was extracted from monocyte and bMDM samples using the RNeasy mini kit (Qiagen) according to the manufacturer’s instructions with on-column DNase digestion. The quality and quantity of the resulting RNA was determined by gel electrophoresis and NanoDrop ND-1000 spectrophotometer (Thermo Scientific). First strand cDNA was reverse transcribed from 0.1-0.5 μg total RNA using oligo(dT) primer and Superscript II (Invitrogen) according to the manufacturer’s instructions. The differentiation markers were amplified by PCR, using ABgene Taq DNA polymerase (Thermo Scientific) following the manufacturer’s instructions. The house-keeping gene glyceraldehyde-3-phosphate dehydrogenase (GAPDH) was also amplified to identify any variation in starting material using previously reported oligonucleotides (Graham et al. 2001).

RT-PCR analysis revealed significant up-regulation of the transcriptional markers; FCGR1A, CD68 molecule, V-maf musculoaponeurotic fibrosarcoma oncogene homolog (avian) (MAF), Triggering receptor expressed on myeloid cells 2 (TREM2), Nuclear receptor subfamily 1, group H, member 3 (NR1H3), Macrophage scavenger receptor 1 (MSR1) and Scavenger receptor class B, member 2 (SCARB2) (Fig. S1). These results are in agreement with the differential expression observed during human monocyte to macrophage differentiation. Furthermore, CEBPD exhibited down-regulation with differentiation, again in agreement with human data. Therefore, the transcriptional data along with the morphology of the bMDM (Fig. S2) confirms macrophage differentiation.


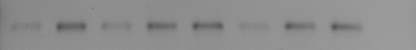

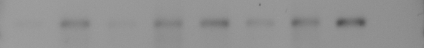

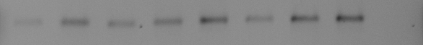

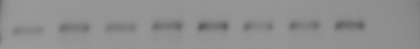

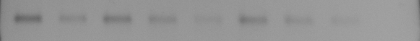

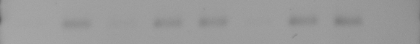

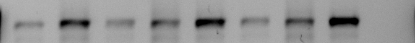

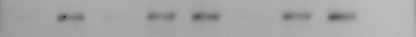

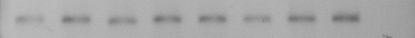


A B C

Mo Mø Mo Mø Mø Mo Mø Mø NC

**GAPDH**

**FCGR1A**

**CD68**

**MAF**

**CEBPD**

**TREM2**

**NR1H3**

**MSR1**

**SCARB2**

**Figure S1.** Summary of RT-PCR results for eight transcriptional markers of human monocyte to macrophage differentiation and GAPDH. Representative results of monocytes and macrophages generated from three Holstein-Friesian cows (A, B and C). bMDM were generated on two separate occasions for animals B and C. NC denotes non-template PCR control.

**Table S1.** Details of the PCR primers and amplicons. F and R denote forward and reverse primers respectively.

|  |  |  |  |  |
| --- | --- | --- | --- | --- |
| **Gene** | **Accession**  **No.** | **Orientation** | **Primer sequence (5’-3’)** | **Product Size (bp)** |
|  |  |  |  |  |
|  |  |  |  |  |
| Glyceraldehyde-3-phosphate | NM_001034034 | F | GATGCTGGTGCTGAGTATGTAGTG | 468 |
| dehydrogenase (GAPDH) |  | R | ATCCACAACAGACACGTTGGGAG |  |
|  |  |  |  |  |
| Fc fragment of IgG, high affinity Ia, | NM_174538 | F | TCTGGTCAACCTGAGCTGTG | 630 |
| receptor (FCGR1A) |  | R | TAAGATGCCAAGGGAGTTGC |  |
|  |  |  |  |  |
| CD68 molecule (CD68) | NM_001045902 | F | AAGAGCCACAGAACCACCAC | 750 |
|  |  | R | GATGATGAGAGGCAGCAAGA |  |
|  |  |  |  |  |
| V-maf musculoaponeurotic fibrosarcoma | AJ815407 | F | TTTGGTCATTGTCAAATGTGG | 261 |
| oncogene homolog (avian) (MAF) |  | R | GCAAGCACATGCTGTATAAGCTAC |  |
|  |  |  |  |  |
| CCAAT/enhancer binding protein | NM_174267 | F | CGTTAGACCCACTTTGATTCC | 508 |
| (C/EBP), delta (CEBPD) |  | R | TGCCCAATACCCACTCTCA |  |
|  |  |  |  |  |
| Triggering receptor expressed on myeloid | NM_001079580 | F | CAGGTGGAGCACAGCATCT | 379 |
| cells 2 (TREM2) |  | R | GCCAAAGGAAGAAGGGTAGAG |  |
|  |  |  |  |  |
| Nuclear receptor subfamily 1, group H, | NM_001014861 | F | TGCTGAGTTTGCCTTGCT | 381 |
| member 3 (NR1H3) |  | R | GAATGTTTGTCCTTCATCTGG |  |
|  |  |  |  |  |
| Macrophage scavenger receptor 1 | NM_001113240 | F | GTAGTTCTCGTGCCCATCA | 596 |
| (MSR1) |  | R | TGTTCCCAATCCTTCAGC |  |
|  |  |  |  |  |
| Scavenger receptor class B, member 2 | NM_001102153 | F | ATGGCACTGGGTGTGTTCTT | 738 |
| (SCARB2) |  | R | CCTATCCTGGGTGAAAGTTGG |  |
|  |  |  |  |  |

**Section 2: Validation of bovine monocyte-derived macrophage (bMDM) purity**

After 7 days the adhered cells were vigorously washed three times with PBS to remove the remaining non-adhered cells. The adherent cells were removed from the plates by incubation with TrypLE Express (Invitrogen) and then resuspended in RPMI-1640 supplemented with 20% FBS. Flow cytometry, using a mouse anti-bovine SIRPA (CD172α) antibody directly conjugated with RPE-Cy5 (AbD Serotec Cat. No. MCA2041C) and a CyAn flow cytometer (Beckman Coulter), confirmed that the macrophage purity exceeded 90%. Fig. S2 illustrates the flow cytometry results for PBMC and resulting bMDM from an example animal.

**SSC**

**SSC**

**FSC**

**FSC**

**CD172a**

**CD172a**

**A**

**B**

**C**

**D**

**97.0%**

R1

**Figure S2.** Representative flow cytometry results for PBMC and resulting bMDM. A and C, flow cytometry FSC v SSC scatterplots illustrating the size and complexity of PBMC and bMDM populations respectively. Debris is gated out of the sample (R1) before determining bMDM purity. B and D, flow cytometry histograms of CD172a levels on the surface of monocytes (subset of PBMC) and gated (R1) bMDM populations respectively. Dotted lines denote unstained samples. Solid lines denote cells stained with anti-bovine SIRPA antibody (Abd Serotec). bMDM purity exceeded 97% in this example.

**Section 3: Quantification of siRNA uptake and cytotoxicity**

The level of siRNA uptake and induced cytotoxicity was measured in bMDM 16 hours post transfection or electroporation with FITC-labelled AllStar negative control siRNA (Qiagen). bMDM were harvested using TrypLE Express, washed with RPMI-1640 supplemented with 20% FBS and resuspended in 1 ml PBS supplemented with 0.5% FBS. Cell toxicity was measured by staining the cells with 1 μM SYTOX Blue dead cell stain (Invitrogen) according to the manufacturer’s protocol. The bMDM were analyzed using a CyAn flow cytometer (Beckman Coulter), the bMDM population was gated, by size and complexity (Fig. S3), and the percentage of FITC labelled and SYTOX Blue labelled bMDM was measured.

**SSC**

**FSC**

**FITC**

**SYTOX Blue**

**A**

**B**

**C**

R1

**80.5%**

**7.0%**

**Figure S3.** Representative flow cytometry results for siRNA uptake and cytotoxicity quantification. A. FSC v SSC scatterplot of untreated bMDM, with gate (R1) around the bMDM population. B & C, histograms illustrating FITC and SYTOX blue staining of gated bMDM population, which indicate siRNA uptake and cytotoxicity respectively. Dotted lines denote non-siRNA treated bMDM stained with SYTOX Blue. Solid lines denote bMDM sample transfected with FITC-labelled siRNA using 2.0 μl DharmaFECT 3.

**References**

Graham, S.P., Brown, D.J., Vatansever, Z., Waddington, D., Taylor, L.H., Nichani, A.K., Campbell, J.D., Adamson, R.E., Glass, E.J., Spooner, R.L., 2001. Proinflammatory cytokine expression by *Theileria annulata* infected cell lines correlates with the pathology they cause *in vivo*. Vaccine 19:2932-2944

Jensen, K., Talbot, R., Paxton, E., Waddington, D., Glass, E.J., 2006. Development and validation of a bovine macrophage specific cDNA microarray. BMC Genomics 7:224.

Lehtonen, A., Ahlfors, H., Veckman, V., Miettinen, M., Lahesmaa, R., Julkunen, I., 2007. Gene expression profiling during differentiation of human monocytes to macrophages or dendritic cells. J Leukoc. Biol. 82:710-720.

Liu, H., Shi, B., Huang, C.C., Eksarko, P., Pope, R.M., 2008. Transcriptional diversity during monocyte to macrophage differentiation. Immunol. Lett. 117:70-80.

Martinez, F.O., Gordon, S., Locati, M., Mantovani, A., 2006. Transcriptional profiling of the human monocyte-to-macrophage differentiation and polarization: new molecules and patterns of gene expression. J Immunol. 177:7303-7311.

Rozen, S., Skaletsky, H.J., 2000. Primer3 on the WWW for general users and for biologist programmers. *In* S. Krawetz and S. Misener (ed.), Bioinformatics Methods and Protocols: Methods in Molecular Biology. Humana Press, Totowa.
